# Multiple Genome Sequences of Lactobacillus pentosus Strains Isolated from Biofilms on the Skin of Fermented Green Table Olives

**DOI:** 10.1128/MRA.01546-18

**Published:** 2019-02-21

**Authors:** Beatriz Calero-Delgado, Antonio J. Pérez-Pulido, Antonio Benítez-Cabello, Antonio M. Martín-Platero, Carlos S. Casimiro-Soriguer, Manuel Martínez-Bueno, Francisco Noé Arroyo-López, Rufino Jiménez Díaz

**Affiliations:** a Departamento de Biotecnología de Alimentos, Instituto de la Grasa, CSIC, Campus de la Universidad Pablo de Olavide, Seville, Spain; b Departamento de Microbiología, Universidad de Granada, Granada, Spain; c Centro Andaluz de Biologia del Desarrollo (CABD, UPO-CSIC-JA), Facultad de Ciencias Experimentales (Área de Genética), Universidad Pablo de Olavide, Seville, Spain; Loyola University Chicago

## Abstract

The draft genome sequences of five Lactobacillus pentosus strains isolated from biofilms on the skin of green table olives are presented here. These genome sequences will assist in revealing the potential probiotic properties of these strains, as the intake of fermented olives implicates the passage of millions of Lactobacillus spp. throughout a consumer’s gastrointestinal tract.

## Abstract (precis)

The draft genome sequences of five Lactobacillus pentosus strains isolated from biofilms on the skin of green table olives are presented here. These genome sequences will assist in revealing the potential probiotic properties of these strains, as the intake of fermented olives implicates the passage of millions of Lactobacillus spp.

## ANNOUNCEMENT

Lactobacillus pentosus is the most important microorganism responsible for the fermentation of olives (1–3). In the past, it was assumed that this species exclusively appeared in a planktonic state (4, 5), but we now know that it makes biofilms on the skin of fermented olives (6, 7). Therefore, billions of L. pentosus cells would be delivered to the human gastrointestinal tract (GIT) with the intake of olives (8). Given the probiotic potential of L. pentosus (9), the fermented olives could be further considered to be a vehicle for the entry of beneficial microorganisms into the GIT. We report here the draft genome sequence of L. pentosus, isolated from biofilms on the skin of traditional fermented olives.

To recover L. pentosus from biofilms, a stomacher method was used (6). Detached biofilms were spread onto de Man-Rogosa-Sharpe (MRS) plates, and isolated colonies were identified at the molecular level as L. pentosus (10). To obtain genomic DNA, a modification of the “salting-out” procedure was followed (11). Genome libraries for DNA sequencing were constructed using a TruSeq DNA PCR-free library preparation kit (Illumina, Inc.), with an insert size of 350 bp. The sequencing process was carried out at Macrogen, Inc. (Seoul, Republic of Korea) using a HiSeq Illumina platform, obtaining paired-end sequencing reads with 2 × 101-bp read lengths. Assembly was performed using Velvet 1.2.10 (12), optimizing parameters with VelvetOptimiser 2.2.5 (12).

The NCBI Prokaryotic Genome Annotation Pipeline (PGAP) (13) was used to annotate the strains, and it was completed using the following protocol: protein-coding genes were predicted using Prodigal version 2.6.3 (14), and then they were functionally annotated by Sma3s v2 using UniProt bacteria (15). To annotate noncoding genes, Infernal 1.1.2 (16) was used with the Rfam database 13.0 (17). To estimate the number of plasmids appearing in each strain, the contig sequences were compared to all the plasmid sequences from Lactobacillus species available in the RefSeq database using BLASTN and 90% for both identity and coverage.

The genomes of all the strains are split into around 100 contigs, having a mean length of 3,795,672 bp, with an estimated G+C content of 45.9%. The numbers of predicted genes were similar in all the cases (Table 1).

**TABLE 1:** Genome information and GenBank accession numbers of five Lactobacillus pentosus strains isolated from biofilms on the skin of fermented Spanish-style green olives

Strain	BioSample accession no.	No. of reads	Avg coverage (×)	Assembly size (bp)	No. of contigs	*N*_50_ (bp)	G+C content (%)	No of protein-coding genes	No. of plasmids	No. of tRNAs	No. of rRNAs
IG8	SAMN10112407	41,135,818	2,790.70	3,791,593	99	312,635	45.91	3,450	6	79	24
IG9	SAMN10112443	34,194,869	957.90	3,787,967	99	278,654	45.91	3,447	6	81	16
IG10	SAMN10112444	36,357,603	972.21	3,811,295	121	98,672	45.95	3,432	7	78	12
IG11	SAMN10112445	41,832,814	2,871.40	3,790,820	107	312,529	45.91	3,448	6	78	20
IG12	SAMN10112446	47,220,444	1,456.30	3,796,685	81	269,556	45.90	3,459	6	80	16

The functional annotation was used to discover genes involved in specific functions, and we also performed a similarity search using BLASTP with a threshold of 80% in both identity and query coverage, using Lactobacillus sequences from the protein database UniProtKB (18). After that, four strains showed two copies of the *luxS* gene, which plays a key role in the synthesis of the universal bacterial communicator autoinductor-2 (19). Also, a high number of genes involved in bacteriocin and exopolysaccharide (EPS) production was found. Interestingly, several genes encoding MucBP proteins, which could play an important role in microbe-eukaryote cell adhesion (20), were also found. Taking into account the importance of all these genes in the probiotic features of lactic acid bacteria, the genome sequences reported here will aid in future research into the probiotic potential of L. pentosus.

### Data availability.

The genome sequences of all the strains have been deposited under NCBI BioProject number PRJNA492883, and the BioSample accession numbers are listed in Table 1. The reads have been deposited in the NCBI Sequence Read Archive (SRA) under the accession numbers SRX5116733 to SRX5116737, and the assemblies have been deposited in GenBank under the accession numbers RDCL00000000, RDCK00000000, RDCJ00000000, RDCI00000000, and RDCH00000000.
